# Aphte géant

**DOI:** 10.11604/pamj.2019.33.273.16157

**Published:** 2019-07-30

**Authors:** Fatima-Zahra Agharbi

**Affiliations:** 1 Centre Hospitalier Régional Tétouan, Tétouan, Maroc

**Keywords:** Aphte, géant, Behcet, Aphthae, giant, Behcet

## Abstract

Aphthae are single or multiple small painful ulcers, preceded by a burning sensation, with a yellow background surrounded by a non-indurated red border, healing usually in 8-10 days. They usually affect the buccal mucosa, but sometimes they are bipolar (orogenital) with possible variants: deep aphthae >1cm, herpetiform aphthae measuring 1-3mm, giants aphthae, miliary aphthae. Benign idiopathic aphtosis is frequent, reactivated by contact with some food (citrus fruit, tomato, walnuts, gruyère). Some drugs can cause aphthoid ulcerations: nonsteroidal anti-inflammatory drugs, nicorandil, alendronate sodium, betablockers, opiate analgesics, savarine, sirolimus, anti-EGFR. Complex aphtosis (at least 3 recurrent episodes of ulcers) can lead to enterocolopathy or celiac disease, sometimes revealing martial or vitamin deficiency (folates, vitamin B12). Bipolar aphtosis is strongly suggestive of Behçet’s disease. We report the case of a 40-year old man presenting for increase in left thigh volume. Ultrasound showed aneurysm of the femoral artery. Clinical examination objectified giant aphthous ulcer in the tongue. Patient interview revealed recurrent genital and buccal aphthae and ophthalmologic examination showed posterior uveitis. The diagnosis of Behçet’s disease was retained and emergency bolus dose of corticosteroids with immunosuppressants (cyclophosphamide) was started.

## Image en médecine

L'aphte est une ulcération douloureuse, de petite taille, précédée d'une sensation de cuisson, unique ou multiple, à fond jaune cerné d'un liseré rouge, non indurée, guérissant habituellement en 8 à 10 jours. Il est fréquent sur la muqueuse buccale, mais parfois bipolaire (orogénital) avec la possibilité de variantes: aphtes profonds >1cm; aphtes herpétiformes: 1 à 3mm; géants; miliaires. L'aphtose idiopathique bénigne est fréquente, réactivée par certains contacts alimentaires (agrumes, tomate, noix, gruyère). Des médicaments sont inducteurs d'ulcérations aphtoïdes: AINS, nicorandil, alendronate monosodique, bêtabloqueurs, analgésiques opiacés, savarine, sirolimus, anti-EGFR. L'aphtose complexe (au moins 3 aphtes récurrents) est parfois inaugurale d'une entérocolopathie ou d'une maladie cœliaque, parfois révélatrice d'une carence martiale ou d'un déficit vitaminique (folates, vitamine B12). Dans l'aphtose bipolaire, il faut rechercher les éléments évocateurs d'une maladie de Behçet. Nous rapportons l'observation d'un homme de 40 ans qui consultait pour une augmentation du volume de la cuisse gauche. L'étude échographique objectivait un anévrysme de l'artère fémorale. L'examen clinique trouvait un aphte géant de la langue. L'interrogatoire trouvait la notion d'aphtose génitale et buccale récidivante et l'examen ophtalmologique objectivait une uveite postérieure. Le diagnostic de maladie de behcet a été retenu un bolus de corticothérapie avec immunosuppresseurs (Cyclophosphamide) a été démarré en urgence.

**Figure 1:**
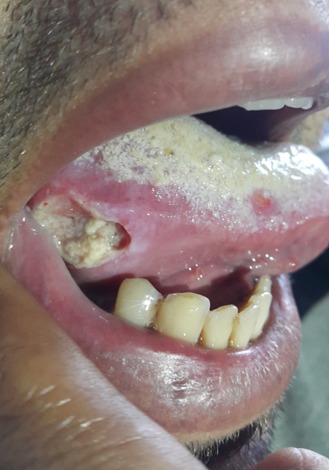
Aphte géant de la langue

